# Antagonistic effect of *Pseudomonas aeruginosa* on *Candida auris*

**DOI:** 10.3389/ffunb.2025.1613244

**Published:** 2025-07-10

**Authors:** Ana Beatriz N. Macedo, Daniele de Figuerêdo Silva, Anthony G. J. Medeiros, Gustavo José Freitas, Murilo Moreira dos Santos, Kelly Ishida, Nalu Teixeira de Aguiar Peres, Daniel Assis Santos, Luana Rossato, Gustavo H. Goldman, Rafael Wesley Bastos

**Affiliations:** ^1^ Departamento de Microbiologia e Parasitologia, Universidade Federal do Rio Grande do Norte, Natal, Rio Grande do Norte, Brazil; ^2^ Departamento de Microbiologia, Universidade Federal de Minas Gerais, Belo Horizonte, Minas Gerais, Brazil; ^3^ Departamento de Microbiologia, Universidade de São Paulo, São Paulo, Brazil; ^4^ Instituto Nacional de Ciência e Tecnologia, Ribeirão Preto, São Paulo, Brazil; ^5^ Laboratório de Pesquisa em Ciências da Saúde, Universidade Federal da Grande Dourados, Dourados, Mato Grosso do Sul, Brazil; ^6^ Faculdade de Ciências Farmacêuticas de Ribeirão Preto, Universidade de São Paulo, Ribeirão Preto, São Paulo, Brazil

**Keywords:** microbial interaction, healthcare-associated infections, fungistatic effect, iron, coculture

## Abstract

**Introduction:**

*Candida (Candidozyma) auris* and *Pseudomonas aeruginosa* are frequently found in hospital environments and on medical equipment, where they commonly colonize and infect hospitalized patients, contributing to healthcare-associated infections (HAIs). Although they share similar ecological niches and may interact, the mechanisms underlying their interspecies communication remain largely unknown.

**Methods:**

This study investigated the *in vitro* interaction between planktonic cells of *C. auris* and *P. aeruginosa* through co-culture experiments in various growth media, with or without iron supplementation. Fluorescence microscopy was employed to assess yeast viability, and the effect of lyophilized, cell-free *P. aeruginosa* supernatants on *C. auris* was also evaluated.

**Results:**

*P. aeruginosa* significantly inhibited the growth of *C. auris*, regardless of the initial microbial concentrations. Growth suppression began after 8 hours of co-culture and persisted for up to 72 hours. Fluorescence microscopy suggested that this antagonistic effect was predominantly fungistatic, as most *C. auris* cells remained viable in the presence of the bacterium. The inhibitory effect was consistent across different culture media, and iron supplementation partially restored *C. auris* growth. Similarly, concentrated cell-free supernatants from *P. aeruginosa* inhibited *C. auris*, further supporting the role of secreted molecules. In this case as well, iron addition partially reversed the inhibitory effect.

**Discussion and conclusion:**

These findings suggest that *P. aeruginosa* produces and secretes molecules with fungistatic activity against *C. auris*, and that this effect is at least partially modulated by iron availability. This discovery provides a foundation for future research into the identity and mechanisms of action of these secreted compounds, as well as the broader clinical implications of microbial interactions during co-colonization or co-infection.

## 1 Introduction

First described in 2009 by Satoh and colleagues, *Candida auris*—now renamed *Candidozyma auris*—has rapidly emerged as a global threat due to its multidrug resistance and ability to persist in hospital environments (Lockhart et al., 2017). This yeast has been included in the World Health Organization (WHO) list of critical priority fungi, underscoring the urgent need for novel antifungal therapies and enhanced public health measures to prevent its spread (World Health Organization, 2022).

*C. auris* transmission occurs primarily through direct contact in hospital settings, where it can colonize patients’ skin and persist in the environment, becoming a significant source of healthcare-associated infections (HAIs) (Alanio et al., 2022). Contaminated medical equipment and hospital surfaces—particularly thermometers and stethoscopes (Govender et al., 2018; Yadav et al., 2021; Didik et al., 2023)—have been identified as key vectors, with outbreaks often traced back to inadequate disinfection practices.

Microorganisms such as *C. auris* coexist with bacteria in human and hospital environments, forming complex and dynamic ecosystems (Peleg et al., 2010; Proctor et al., 2021). A noteworthy pathogen in healthcare settings is *Pseudomonas aeruginosa*, a highly antibiotic-resistant bacterium capable of long-term persistence in hospital environments, similar to *C. auris* (Grainha et al., 2020).

*P. aeruginosa* is a Gram-negative, non-glucose fermenting, motile, oxidase-positive bacillus that thrives in a wide range of environments, from aquatic and terrestrial ecosystems to plants, animals, and humans (Grainha et al., 2020). It is notorious for its ability to form biofilms on abiotic surfaces, such as medical implants and industrial equipment, as well as within the human host, particularly in the respiratory tracts of individuals with cystic fibrosis (Thi et al., 2020). Although capable of infecting immunocompetent individuals, it is most prevalent in immunocompromised or hospitalized patients undergoing broad-spectrum antibiotic therapies, making it a leading cause of HAIs, often affecting the respiratory and urinary tracts, as well as the bloodstream (Mielko et al., 2019).

In this context, a case report of *P. aeruginosa* infection in a patient colonized by *C. auris* (Proctor et al., 2021) has been documented, along with metagenomic data showing that both microorganisms can colonize the skin of nursing home residents (Proctor et al., 2025). These findings suggest that *P. aeruginosa* may come in contact and directly interact with *C. auris* in hospital environments and/or within the host, potentially altering the dynamics of these pathogens in such settings.

Although the implications of the interaction between *C. auris* and *P. aeruginosa* are still poorly understood, emerging evidence suggests that *P. aeruginosa* exhibits antimicrobial and antibiofilm activities against a wide variety of other fungi, including *Aspergillus fumigatus* (Bastos et al., 2022), *Cryptococcus* spp. (Peres-Emídio, 2020), and *Candida albicans (* Trejo-Hernández et al., 2014). In the case of *P. aeruginosa*–C*. albicans*, the interaction involves molecular communication mediated by compounds produced in response to mutual stimulation between the microorganisms. The yeast secretes ethanol, which induces *P. aeruginosa* to synthesize and release toxic phenazines. These phenazines, in turn, promote even greater ethanol production by *C. albicans*, resulting in the functional collapse of mitochondria (Greenberg et al., 1999; Morales et al., 2013; Lindsay et al., 2014; d’Enfert et al., 2021). Furthermore, phenazines and other metabolites secreted by *P. aeruginosa* can interfere with iron homeostasis in *C. albicans* and other fungi, leading to growth arrest (Purschke et al., 2012; Peres-Emídio, 2020; Proctor et al., 2025).

Given the complexity of these interaction and their potential impact on co-colonization, the goal of this study is to evaluate the *in vitro* interaction between *C. auris* and *P. aeruginosa*, in comparison with the interaction with *C. albicans*, to identify factors that may influence these dynamics.

## 2 Materials and methods

### 2.1 Microorganisms

*C. auris* isolates from different origins were used: *C. auris* 467/15 (Venezuela) (Calvo et al., 2016), *C. auris* 136/18 (South Africa) (Magobo et al., 2014), *C. auris* 139/18 (Spain) (Ruiz-Gaitán et al., 2018), *C. auris* 138/18 (South Korea) (Lee et al., 2011), *C. auris* 140/18 (England) (Schelenz et al., 2016), and *C. auris* CBS 10913 (Japan) (Satoh et al., 2009). The reference strain *C. albicans* ATCC (American Type Culture Collection) 90028 and the clinical isolates of *P. aeruginosa* Pa14 (Rahme et al., 1997), Pa01 (Mikkelsen et al., 2011; Wettstadt et al., 2019), and Pak (Saliba et al., 2005) also were used. All strains were maintained in BHI medium (brain heart infusion; Kasvi, Brazil) supplemented with 10% glycerol at −80°C until use.

### 2.2 Interaction between *C. auris* and *P. aeruginosa* using the spot-on-the-lawn method

The spot-on-the-lawn antagonism method was performed according to Harris et al. (1989), with the modifications introduced by Peres-Emidio et al. (2022). Initially, 10 μL of *P. aeruginosa* Pa14, Pa01, and Pak (at 10^6^ colony-forming units [CFU]/mL), previously grown for 16 hours in BHI at 37°C, were spotted onto the center of Petri dishes containing BHI agar and incubated at 37°C for 18 hours.

The bacterial cells were then inactivated with 1 mL of chloroform vapor added to the lids of each plate. After 30 minutes of incubation at room temperature, the plates were left uncovered until the chloroform completely evaporated. Subsequently, 3 mL of semi-solid YPD medium (1% yeast extract, 2% peptone, 2% dextrose, and 0.75% agar), with 1×10^5^ CFU/mL of either *C. albicans* ATCC90028 or *C. auris* 467/15, was poured over the solid BHI medium containing the inactivated bacterial spot. After incubation for 48 hours at 37°C, the inhibition zones formed on the fungal cell lawn were visually measured. The *C. auris* and *C. albicans* cultures used in this assay were obtained from previous cultivation in YPD liquid medium for 24 hours.

### 2.3 Interaction between *C. auris* and *P. aeruginosa* using the co-culture in liquid medium method

Yeast inhibition was also evaluated in the co-cultivation of *C. auris* 467/15 with *P. aeruginosa* Pa14 in liquid medium (Peres-Emidio et al., 2022). Co-cultures and monocultures of *C. auris* and *P. aeruginosa* were prepared using different bacterial inocula (1×10^5^, 10^6^, and 10^7^ CFU/mL) and a fixed fungal inoculum of 1×10^5^ CFU/mL. The experiment was conducted in 1.5 mL tubes containing 1 mL of RPMI-1640 (Roswell Park Memorial Institute) medium (Gibco, USA) buffered with 34.5 g of MOPS (3-(N-morpholino) propanesulfonic acid) (Sigma-Aldrich, USA), with the pH adjusted to 7.0–7.2.

After incubation at 37°C for 24 hours, aliquots were plated on YPD medium with 0.2 mg/L of chloramphenicol. The plates were incubated at 37°C for 48 hours for CFU counting. The results were expressed in log CFU/mL, comparing fungal growth in monocultures and co-cultures (Peres-Emídio, 2020). The number of input cells in each case was similarly determined to ensure equivalent initial inocula of the bacterium and yeast.

As a control, the interaction between *Candida albicans* 90028 and *P. aeruginosa* Pa14 was included in all experiments, following the same protocol.

To broaden the findings obtained with the *C. auris* 467/15 strain, additional interaction assays in liquid medium were performed using *C. auris* isolates from diverse geographic origins: *C. auris* 136/18, 138/18, 139/18, 140/18, and CBS 10913. In these assays, a fungal inoculum of 1×10^5^ CFU/mL and a bacterial inoculum of 1×10^6^ CFU/mL of *P. aeruginosa* Pa14 were used.

### 2.4 Kinetics of the interaction between *P. aeruginosa* and *C. auris*

A microbial kinetics experiment investigated the interaction between *P. aeruginosa* Pa14 and *C. auris* 467/15 over time. *P. aeruginosa* (1×10^6^ CFU/mL) was co-cultured with *C. auris* (1×10^5^ CFU/mL) in 1 mL of RPMI medium at 37°C—monoculture controls of each microorganism were also included. Samples were collected at 0, 4, 8, and 12 hours, and aliquots were plated on YPD agar supplemented with 0.2 mg/L chloramphenicol to determine yeast CFUs. To extend these findings, a subsequent experiment was conducted considering longer intervals: 0, 12, 24, 48, and 72 hours. At each time, aliquots were collected and plated to quantify bacterial CFUs on Cetrimide agar (Kasvi, Brazil) and yeast CFUs on YPD agar with 0.2 mg/L chloramphenicol.

### 2.5 LIVE/DEAD staining by fluorescent microscopy

Cell viability resulting from the interaction between *C. auris* 467/15 or *C. albicans* ATCC 90028 and *P. aeruginosa* Pa14 was assessed via fluorescence microscopy, following a modified protocol based on Sun et al. (2016). Co-cultures and monocultures were prepared in liquid RPMI medium as previously described, using inocula of 1×10^6^ CFU/mL for *P. aeruginosa* and 1×10^5^ CFU/mL for the fungi. After 72 hours of incubation at 35°C, the microtubes were centrifuged, the supernatant was discarded, and 300 μL of LIVE/DEAD (SYTO 9/PI) fluorescent staining solution—containing 5 μM SYTO 9 and 10 μg/mL propidium iodide (PI) (both from Invitrogen, USA), diluted in phosphate-buffered saline (PBS)—was added to the pellet. This solution stains viable cells green (SYTO 9) and dead cells red (PI).

Samples were incubated with the stain for 30 minutes at 35°C, protected from light. After that, the cells were washed twice with 300 μL of PBS, which was also used as the final resuspension volume. Finally, 20 μL of each sample was placed on a microscope slide, covered with a coverslip, and examined under a fluorescence microscope (EVOS FL Cell, USA).

Microscopic images from the co-culture and monoculture groups were processed using ImageJ software (https://imagej.net/ij/) to quantify live cells. Transmitted light (Trans) images, captured without a fluorescence filter, were converted to 8-bit, followed by thresholding and post-thresholding refinement steps, and used for automatic cell counting. RGB images, generated from the fluorescence emitted by cells labeled with IP and SYTO 9, were processed by selecting the red and green channels to generate a composite image, in which automatic cell labeling and manual cell counting were performed.

Results were expressed as total cell count, number of live cells, and percentage of live cells. Total cell counts from monocultures (*C. auris* 467/15 or *C. albicans* ATCC 90028) and co-cultures (*C. auris* 467/15 + *P. aeruginosa* Pa14/*C. albicans* ATCC 90028 + *P. aeruginosa* Pa14) were obtained from images captured under transmitted light (no filter). The number of live cells was determined by subtracting the number of dead cells (PI-labeled) from the total cell count in each condition. This value was cross-validated by comparison with the number of SYTO 9-labeled cells (green fluorescence). The percentage of live cells was calculated using the formula:


$$\text{\% live cells}:\;\frac{\left(\text{total number of cells}-\text{PI labeled dead cells}\right)\;}{\text{total number of cells}}\text{x }100$$

All counts were performed on five replicates derived from three independent experimental groups (biological triplicates) for both monocultures and co-cultures.

### 2.6 Co-culture in different media

To investigate whether nutrient availability influences the bacterium-yeast interaction, co-cultures of *P. aeruginosa* Pa14 and *C. auris* 467/15, along with the respective controls, were performed in the following media: YPD, RPMI supplemented with 2% (w/v) glucose, 2-fold concentrated RPMI (RPMI 2x), and RPMI supplemented with FeSO₄ (at 30, 120, 240, 480, 960, and 1920 μM). The results were compared with those obtained using standard RPMI, following the previously described liquid co-culture protocol, using a 10:1 bacterium-to-yeast ratio.

### 2.7 Cultivation of *C. auris* in cell-free supernatant from *P. aeruginosa* culture

*P. aeruginosa* Pa14 was cultured individually for 24 or 72 hours at 37°C. Following incubation, the cultures were centrifuged, and the supernatants were filtered through a 0.22 μm membrane to obtain the cell-free supernatant (CFS). The CFS was then mixed with RPMI in a 3:1 ratio (three parts CFS to one part 4-fold concentrated RPMI) and used to culture *C. auris* 467/15 at a final concentration of 1×10^5^ CFU/mL. As a control, *C. auris* was also cultured in a solution composed of three parts 0.85% NaCl (w/v) and one part 4-fold concentrated RPMI. After 24 hours of incubation at 37°C, yeast cells were plated on YPD agar, and CFUs were counted and compared across treatment groups.

### 2.8 Antifungal activity of lyophilized cell-free supernatant of *P. aeruginosa*

*P. aeruginosa* Pa 14 (1×10^6^ cells/mL) was cultured for 24 hours in 10 mL of RPMI medium at 37°C. After incubation, the culture was centrifuged and filtered to obtain the CFS. The CFS and a control containing only RPMI were frozen and lyophilized for 48 hours, then reconstituted in 1 mL of RPMI. A portion of the reconstituted supernatant was supplemented with varying concentrations of ferrous sulfate (FeSO_4_ at 30, 480, and 1920 μM). Subsequently, 100 μL of *C. auris* 467/15, adjusted to 1×10^5^ cells/mL, was added to 96-well plates with 100 μL of the reconstituted supernatant. The experimental groups comprised lyophilized cell-free supernatant (lCFS) of *P. aeruginosa* (lCFS_Pa_) and lCFS of *P. aeruginosa* supplemented with FeSO_4_ (lCFS_Pa_ + FeSO_4_). Lyophilized RPMI and fresh RPMI were used as growth control groups.

The plates were incubated at 37°C, and visual assessments were conducted at 24, 48, and 72 hours. After 72 hours, aliquots from each experimental group were plated on YPD agar medium to quantify fungal viability.

### 2.9 Statistical data analysis

The results were subjected to statistical analysis using PRISM 8.0 software (GraphPad Inc., San Diego, CA, USA) to assess significant differences among the experimental groups. One-way or two-way ANOVA was applied, followed by Tukey’s post-test or t-test at a 95% significance level. All experiments were performed independently at least three times.

## 3 Results

### 3.1 *P. aeruginosa* inhibits *C. auris* growth similarly to *C. albicans*

**Figure 1:**
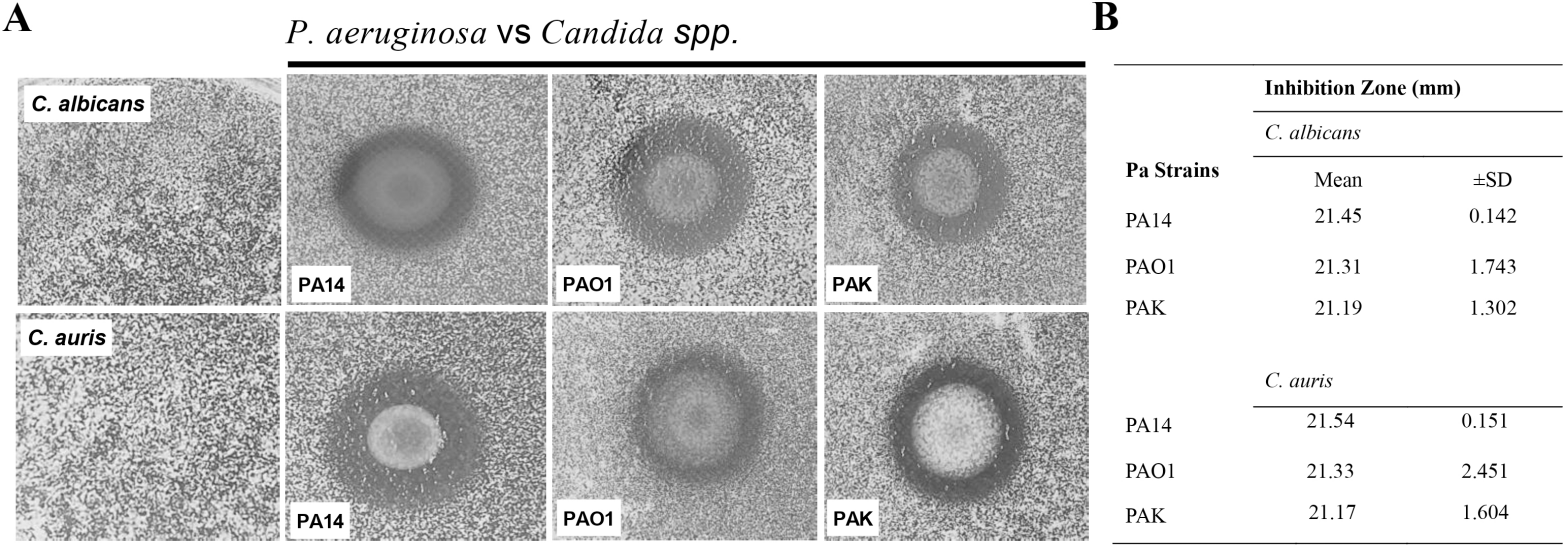
Inhibition of *Candida* spp. growth by *P. aeruginosa*. **(A)** Inhibition of *Candida* spp. growth observed in the spot-on-the-lawn assay with PA14, PAO1, and PAK. The images show the inhibition zone produced by each bacterium strain. **(B)** Inhibition zone measurements after interaction between *P. aeruginosa* and *C*. *auris* or *C*. *albicans*. Data are representative of three independent experiments.

The interaction between *C. auris* and *P. aeruginosa* was analyzed using the spot-on-the-lawn method and co-cultivation in a liquid medium. The spot-on-the-lawn assay revealed inhibition zones caused by products secreted by *P. aeruginosa* strains (PA14, PAO1, and PAK), demonstrating that *C. auris* (467/15) is inhibited by this bacterium (**Figure 1**). The inhibition zones averaged 21.45 mm for strain PA14 (range = 20.4–21.5 mm), 21.31 mm for PAO1 (19.1–22.7 mm), and 21.19 mm for PAK (18.5–20.6 mm) (**Figure 1**). A similar effect was observed in the interaction with *C. albicans* (**Figure 1**).

Although no statistically significant difference was detected among the bacterial strains (**Figure 1**), *P. aeruginosa* PA14 was selected for the subsequent experiments, as it has been widely used in fungal interaction studies (Peres-Emídio, 2020; Bastos et al., 2022).

**Figure 2:**
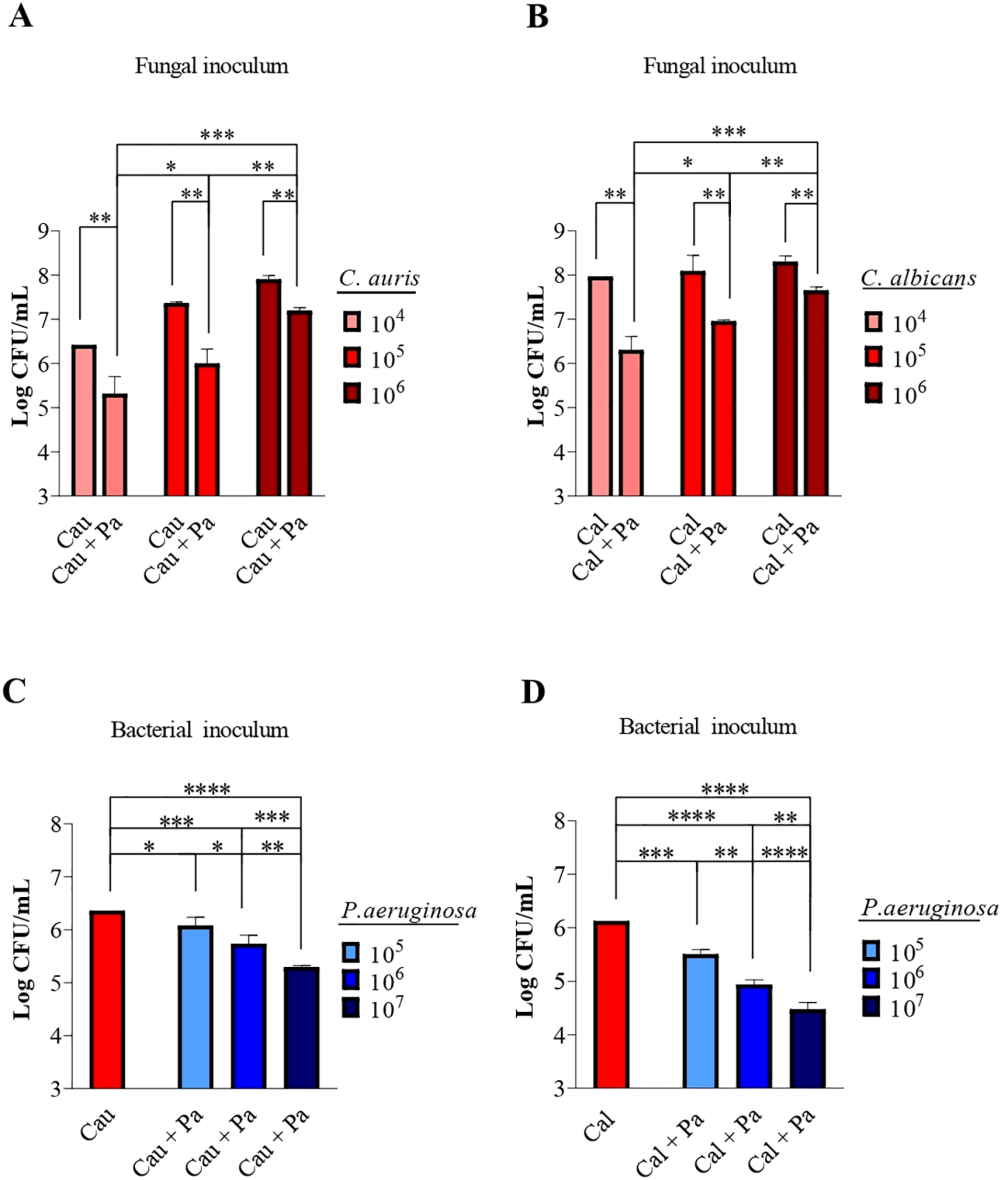
*Candida* spp. inhibition by *P. aeruginosa* in a liquid medium. Growth of *C*. *auris* (Cau) **(A)** and *C*. *albicans* (Cal) **(B)** at different concentrations, in isolation and co-culture with *P. aeruginosa* (Pa). Growth of *P. aeruginosa* at different concentrations, in isolation and co-culture with *C*. *auris* **(C)** and *C*. *albicans* **(D)**. Data are representative of three independent experiments. Statistical analysis was performed using ANOVA followed by Tukey’s post-test. *p<0.05; **p<0.01; ***p<0.001; ****p<0.0001.

Co-cultivation in a liquid medium was carried out to gain deeper insights into the phenomenon of antagonism. In the first step, varying concentrations of *C. auris* (10^4^, 10^5^, and 10^6^ CFU/mL) were co-cultured with a fixed concentration of *P. aeruginosa* (1×10^6^ CFU/mL) in RPMI liquid medium. As shown in **Figure 2A**, *P. aeruginosa* significantly inhibited yeast growth—by nearly 1 log cycle (10-fold) compared to *C. auris* grown in isolation—regardless of the fungal inoculum concentration. Notably, the inhibition was most pronounced at the lowest yeast inoculum (10^4^ CFU/mL). This result parallels the inhibitory effect observed for *C. albicans* when co-cultured with *P. aeruginosa* (**Figure 2B**).

Next, the yeast inoculum was kept constant (1×10^5^ CFU/mL) while the initial bacterial concentration varied (10^5^, 10^6^, and 10^7^ CFU/mL). The results showed that higher bacterial inocula led to more significant inhibition of *C. auris* (**Figure 2C**) and *C. albicans* (**Figure 2D**).

### 3.2 Co-cultivation with *P. aeruginosa* inhibits other *C. auris* strains

To investigate whether other *C. auris* isolates exhibit similar interaction behavior with *P. aeruginosa*, different *C. auris* strains were co-cultured in a liquid medium with the bacterium (1×10^5^ CFU/mL of yeast and 1×10^6^ CFU/mL of bacterium). Yeast concentrations were measured after 24 hours of co-cultivation.

**Figure 3:**
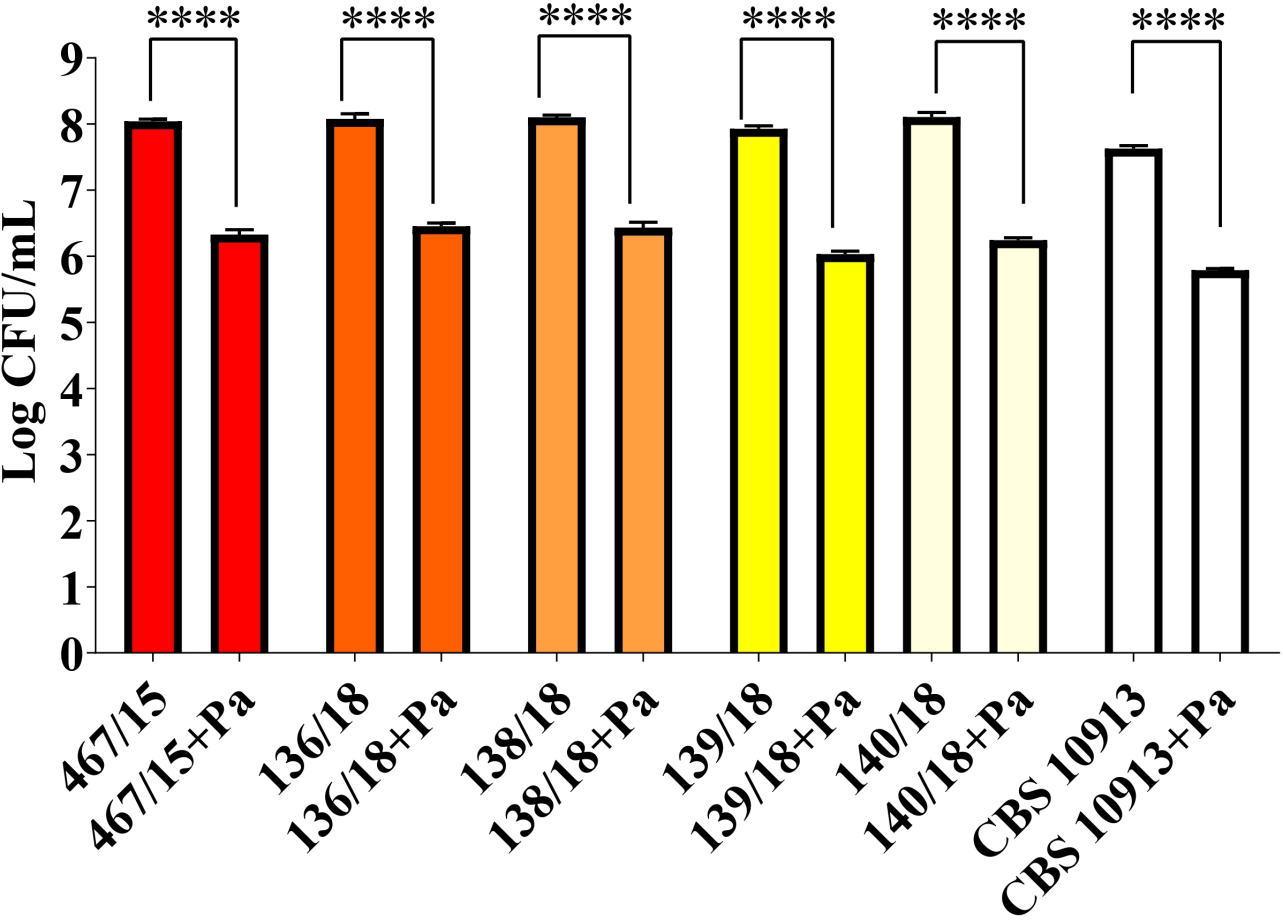
*P. aeruginosa* (Pa) consistently inhibited the growth of different *C. auris* strains (including 467/15). Data are representative of three independent experiments. Statistical analysis was performed using Student’s t-test. ****p<0.0001.

**Figure 3** shows that the growth of all six *C. auris* strains (467/15, 136/18, 138/18, 139/18, 140/18, and CBS 10913) was significantly inhibited by *P. aeruginosa* compared to monoculture. This indicates that *P. aeruginosa* exerts a consistent antagonistic effect against multiple *C. auris* strains.

### 3.3 Kinetics of *C. auris* inhibition by *P. aeruginosa*

**Figure 4:**
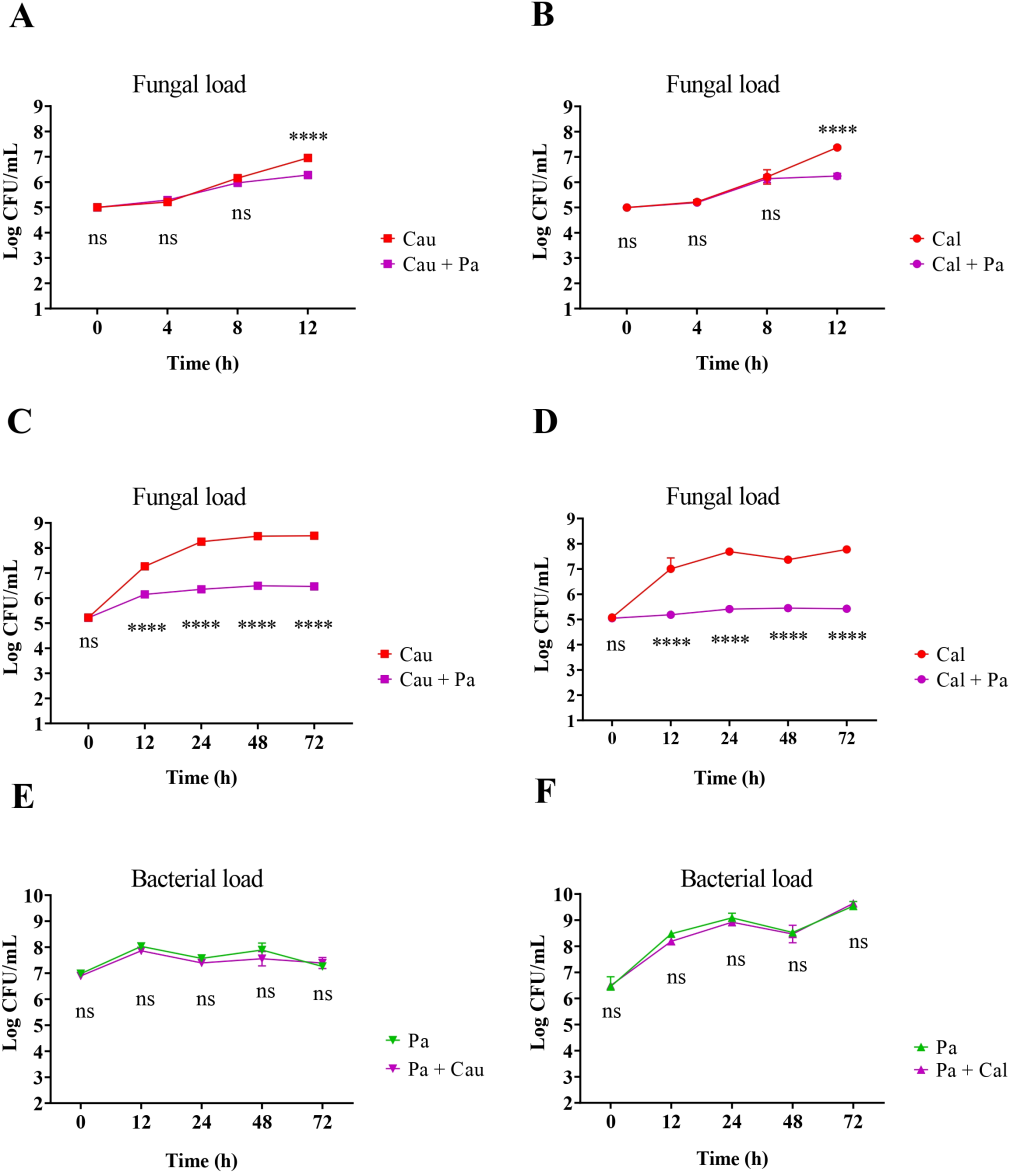
Growth kinetics of *C. auris*, *C. albicans*, and *P. aeruginosa* in monoculture and co-culture. *P. aeruginosa* (Pa) significantly affected the growth of *C. auris* (Cau) and *C. albicans* (Cal) between 8 and 12 hours **(A, B)**. Growth inhibition persisted for up to 72 hours in both yeast species **(C, D)**. However, *P. aeruginosa* growth remained unaffected by the presence of either *C. auris* or *C. albicans* **(E, F)**. Statistical analysis was performed using a two-way ANOVA. ****p<0.0001; ns, not significant (comparison between monoculture and co-culture at the same time points).

After demonstrating that *P. aeruginosa* consistently inhibits different *C. auris* strains during co-cultivation, additional experiments were performed using *C. auris* 467/15. First, the kinetics of the interaction were investigated to establish the time point of maximum inhibition. **Figures 4A, B** show a substantial negative effect of *P. aeruginosa* on *C. auris* growth, which became significant between 8 and 12 hours of co-cultivation.

Further analysis revealed that the inhibition of *C. auris* by *P. aeruginosa* persisted steadily throughout the 72-hour evaluation period, with no statistically significant changes over time (**Figure 4C**). Interestingly, the fungal load in contact with the bacterium remained stable at approximately a 5-log cycle during the experiment (**Figure 4C**). A similar pattern was observed for *C. albicans*, which exhibited comparable inhibition under the same conditions (**Figure 4D**). Moreover, no significant differences were observed in bacterial concentrations between monoculture and co-culture conditions with either *C. auris* (**Figure 4E**) or *C. albicans* (**Figure 4F**), indicating that these yeasts were unable to inhibit the bacterium growth significantly.

### 3.4 Evaluation of the fungistatic effect of *P. aeruginosa* on *C. auris*

As no significant changes in *C. auris* inhibition were observed over time during co-cultivation with *P. aeruginosa* from 12 to 72 hours (**Figure 4C**), we hypothesized that the bacterial effect might be fungistatic. To test this, *C. auris* and *P. aeruginosa* were cultured together and separately for 72 hours. The cells were then collected and stained with fluorescent markers to quantify total and live cells and calculate the live cell percentage.

**Figure 5:**
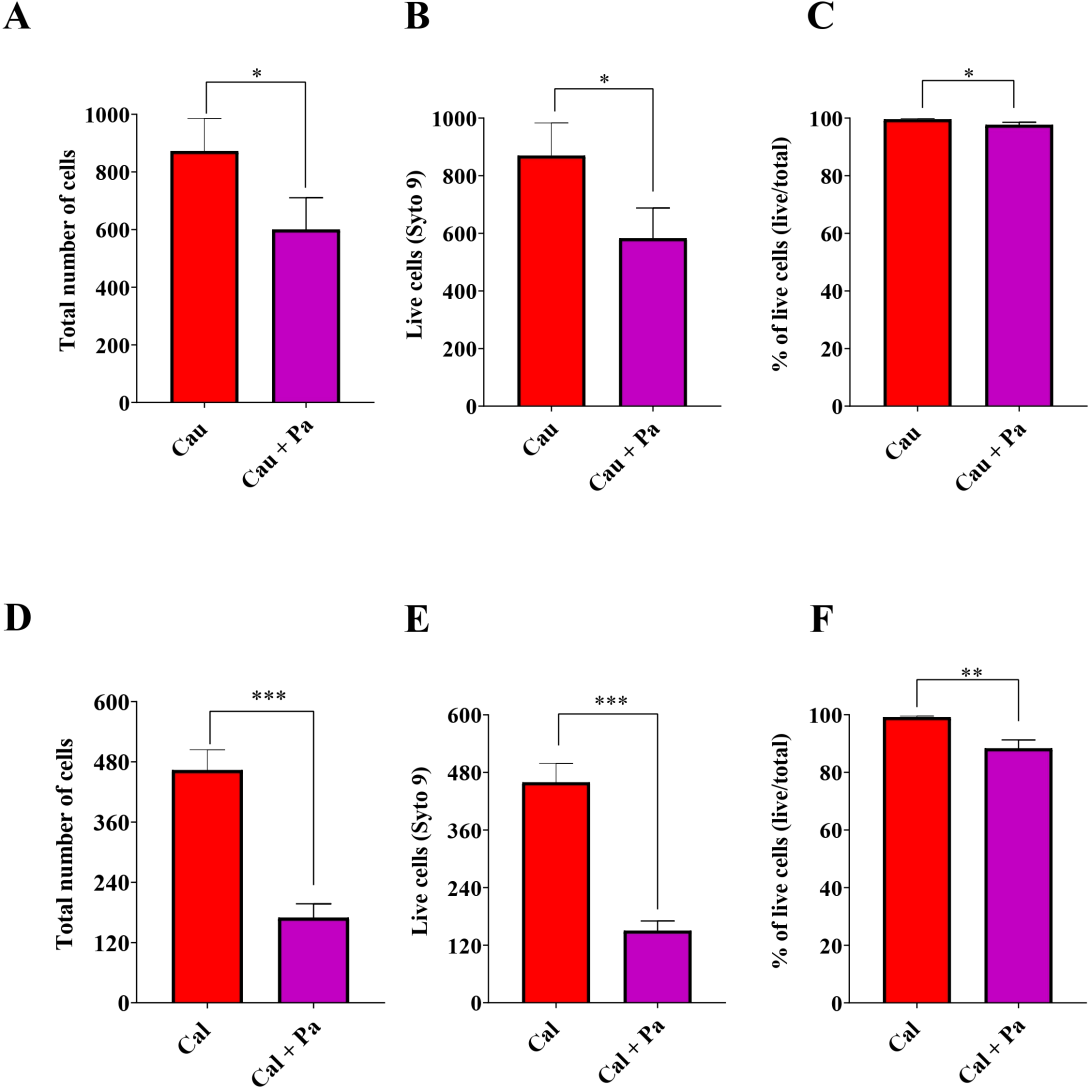
*P. aeruginosa* exerts a fungistatic effect on *Candida* spp. LIVE/DEAD (SYTO9/PI) staining and fluorescence microscopy were performed after 72 hours of incubation. **(A, D)** Total cell counts of *C. auris* (Cau) or *C. albicans* (Cal) grown in monoculture or co-culture with *P. aeruginosa* (Pa), showing reduced fungal cell counts in the presence of the bacterium. **(B, E)** Live yeast cells (labeled with SYTO 9 and PI), highlighted after RGB image processing to quantify live and dead populations, also decreased under co-culture. **(C, F)** The percentage of live yeasts in monoculture versus co-culture indicates that, although total and live cell counts are reduced in the latter, the majority of cells remain live, indicating a fungistatic effect. Data are representative of three independent experiments. Statistical analysis was performed using Student’s t-test. *p<0.05; **p<0.01; ***p<0.001.

A significant reduction in both total and live *C. auris* cell counts was observed in the co-culture with *P. aeruginosa* compared to monoculture (**Figures 5A, B**). Additionally, a slight decrease in the percentage of live cells was detected (monoculture mean = 99.58% vs. co-culture mean = 97.67%) (**Figures 5C**). Notably, the number of *C. auris* cells in co-culture reduced to 36% compared to monoculture; yet, 97% of these cells remained live after 72 hours of co-cultivation with *P. aeruginosa*. This slight reduction in viability, in contrast to the pronounced decline in total and live cell counts, suggests that *P. aeruginosa* exerts a fungistatic rather than fungicidal effect on *C. auris*, as the majority of yeast cells remain alive but fail to proliferate in the presence of the bacterium (**Figures 5A–C**).

A similar experiment conducted with *C. albicans* yielded comparable results. After 72 hours, a reduction in both total and viable cell counts was evident, along with a slight decrease in the percentage of viable cells, further supporting a fungistatic effect under the influence of *P. aeruginosa* (**Figures 5D–F**).

### 3.5 *C. auris* growth inhibition by *P. aeruginosa* is consistent in different culture media

**Figure 6:**
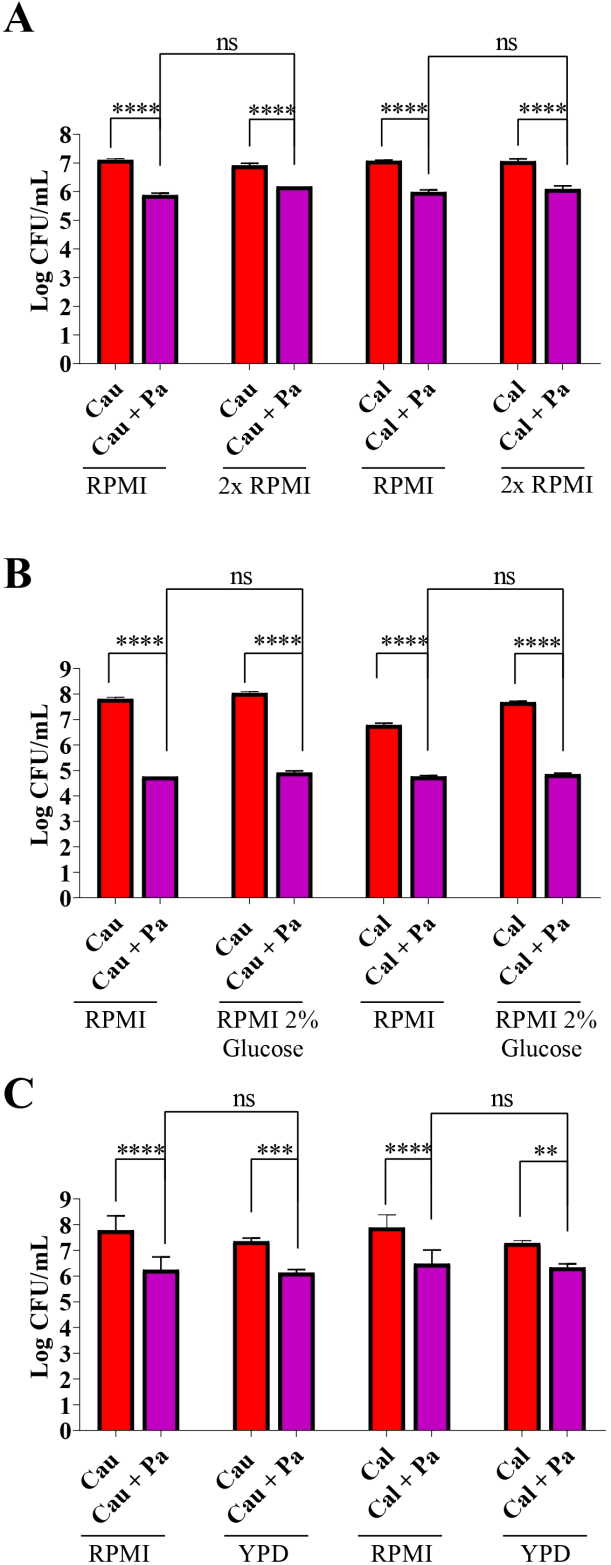
Growth inhibition of *Candida* spp. by *P. aeruginosa* is consistent across different media with varying nutritional compositions. *C*. *auris* (Cau) and *C*. *albicans* (Cal) were cultured alone or co-cultured with *P. aeruginosa* (Pa) in standard RPMI and compared with 2-fold RPMI **(A)**, RPMI + 2% glucose **(B)**, and YPD **(C)**. The results show that the bacterium inhibits yeast growth independently of the culture medium. Data are representative of three independent experiments. Statistical analysis was performed using Student’s t-test. **p<0.01; ***p<0.001; ****p<0.0001; ns, not significant.

To evaluate whether different culture media and their compositions influence the interaction between *C. auris* and *P. aeruginosa*, we tested 2-fold concentrated RPMI, RPMI supplemented with 2% glucose, and liquid YPD, using standard RPMI as the reference condition. The inhibition of *C. auris* by *P. aeruginosa* remained consistent regardless of the medium used (**Figures 6A–C**). Moreover, the presence of additional nutrients, such as glucose in RPMI or the components of YPD, did not alter the inhibition pattern, showing no significant difference compared to the standard (**Figures 6A–C**).

### 3.6 Iron partially restores *C. auris* growth in co-culture

**Figure 7:**
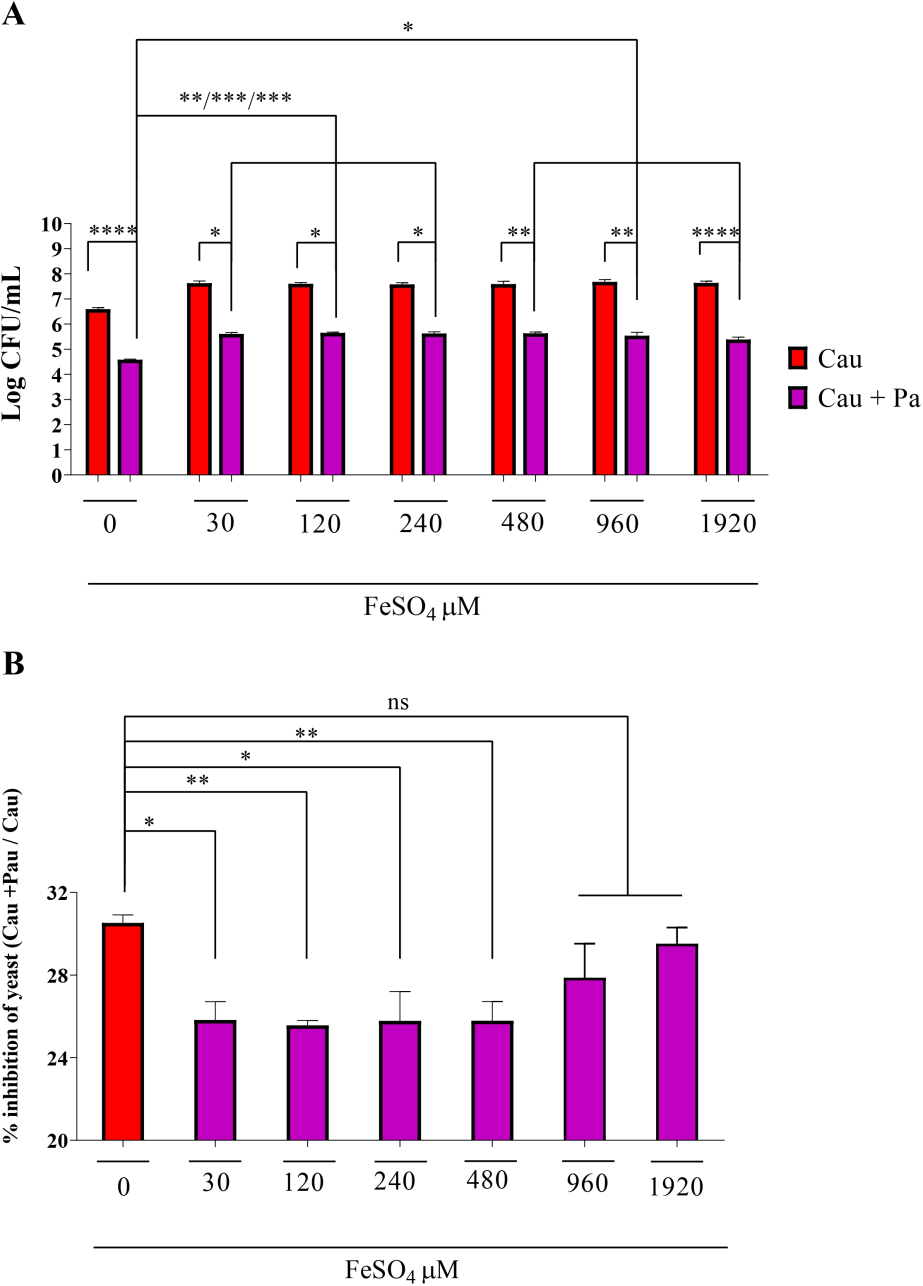
FeSO_4_ partially restores *C*. *auris* growth by reducing the inhibitory effect caused by *P. aeruginosa* (Pa). **(A)** Different concentrations of FeSO_4_ (30, 120, 240, 480, 960, and 1920 μM) were tested; concentrations above 30 μM showed no additional effect on fungal growth restoration. **(B)** Significant reduction in growth arrest of *C*. *auris* in coculture for iron concentrations between 30 and 480 μM; however, higher concentrations (960 and 1920 μM) did not promote additional effects. Data are representative of three independent experiments. Statistical analysis was performed using ANOVA and Tukey’s post-test. *p<0.05; **p<0.01; ***p<0.001; ****p<0.0001; ns, not significant.

The effect of iron supplementation on *C. auris* inhibition by *P. aeruginosa* was evaluated using RPMI medium supplemented with varying concentrations of FeSO_4_. Iron significantly alleviated the inhibitory effect at all tested concentrations, with no further improvement observed beyond 30 μM (**Figure 7A**). It is important to note that the growth of *C. auris* in monoculture also was substantially enhanced by FeSO_4_ supplementation (statistical analysis not shown).

To further confirm the impact of iron on the antagonistic effect of *P. aeruginosa* and to control for the growth enhancement caused by iron supplementation alone, we calculated the percentage of inhibition for each treatment by comparing *C. auris* growth in monoculture versus co-culture with *P. aeruginosa* in the presence and absence of FeSO_4_ (**Figure 7B**). The results confirmed that iron concentrations between 30 and 480 μM significantly reduced growth inhibition, while higher concentrations did not produce additional benefits.

Overall, these findings indicate that Fe^2+^ supplementation partially restores *C. auris* growth in co-culture with *P. aeruginosa* compared to monoculture conditions.

### 3.7 *P. aeruginosa* cell-free supernatant (CFS) inhibits *C. auris* in a concentration-dependent manner

**Figure 8:**
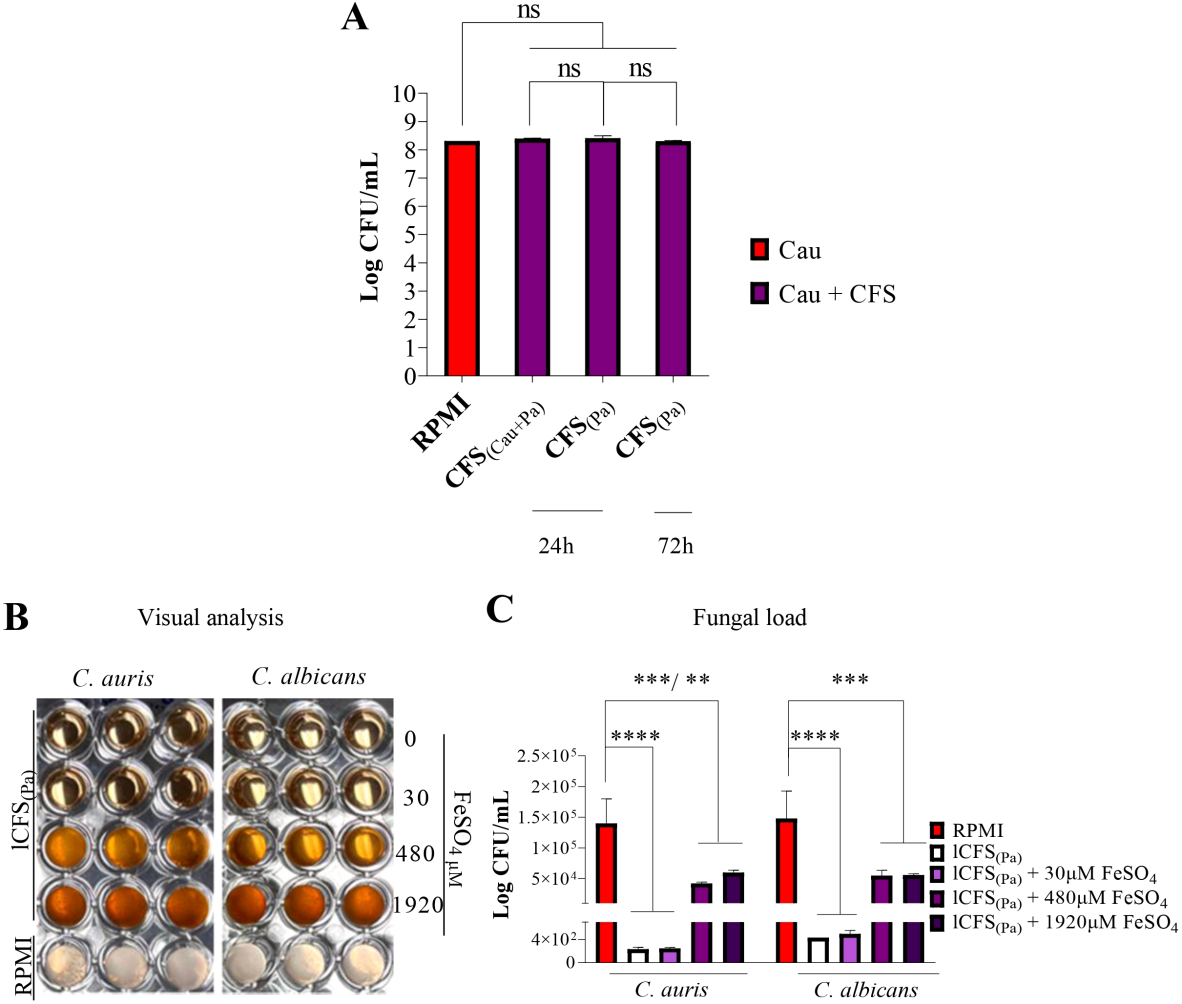
The cell-free supernatant (CFS) of *P. aeruginosa* did not inhibit *Candida* spp. growth unless concentrated. CFS from *P. aeruginosa* (Pa) cultured for 24 or 72 hours and CFS from *P. aeruginosa* co-cultured with *C*. *auris* did not significantly affect *C*. *auris* (Cau) growth **(A)**. In contrast, CFS from *P. aeruginosa* concentrated by lyophilization (lCFS_(Pa)_) inhibited the growth of both *C*. *auris* and *C*. *albicans*. **(B)** Representative images show fungal growth inhibition in RPMI (control), RPMI + lCFS_(Pa)_, and RPMI + lCFS_(Pa)_ + FeSO_4_. **(C)** CFU/mL count of *Candida* spp. growth. *Candida* growth was reduced in RPMI + lCFS_Pa_ compared to RPMI alone. Supplementation with higher concentrations of FeSO_4_ (480 and 1920 μM) increased the growth in RPMI + lCFS_(Pa)_, although not to the same extent as in the control. Statistical analysis was performed using ANOVA followed by Tukey’s post-test. **p<0.01; ***p<0.001; ****p<0.0001; ns, not significant.

Since *P. aeruginosa* inhibits the growth of *C. auris* even in the absence of viable bacterial cells (spot-on-the-lawn assay), it is likely that the bacterium produces and secretes molecules with fungistatic activity. To investigate whether this inhibition is mediated by secreted compounds, cell-free supernatant (CFS) was obtained after 24 hours of *P. aeruginosa* growth and used to culture *C. auris* and *C. albicans*. Unlike in co-culture conditions, the crude CFS did not inhibit yeast growth (**Figure 8A**). Similarly, the growth of *P. aeruginosa* was unaffected when cultured in crude CFS derived from *C. albicans* or *C. auris* cultures (**Figure 8A**).

The potential influence of bacterial culture duration before filtration was also evaluated. When *P. aeruginosa* was cultivated for an extended time (72 hours) before supernatant collection, the resulting CFS_Pa_ also failed to inhibit *Candida* growth (**Figure 8A**). To test whether the presence of the yeast is required to trigger the production of antagonistic metabolites, a co-culture was established for 24 hours, and the resulting CFS_(Cau + Pa)_ was subsequently used to culture *C. auris* for an additional 24 hours. Neither in this case was the growth of *C. auris* inhibited by the crude CFS_(Cau + Pa)_ (**Figure 8A**).

The inhibitory effect of *P. aeruginosa* CFS on *C. auris* was reassessed using a 10-fold concentrated supernatant obtained by lyophilization (lCFS_Pa_). In 96-well plate assays, visual analysis confirmed that lCFS_Pa_ completely inhibited *C. auris* growth (**Figure 8B**). After 72 hours of incubation, lCFS_Pa_ effectively suppressed the yeast growth compared to RPMI alone, with similar inhibitory effects observed for *C. albicans* (**Figures 8B, C**).

To investigate the role of iron in modulating this interaction, lCFS_Pa_ was reconstituted in RPMI and supplemented with FeSO_4_. At 30 μM, iron supplementation failed to reverse the inhibitory effect of lCFS_Pa_. However, higher concentrations (480 and 1920 μM) significantly increased yeast growth compared to cultures with lCFS_Pa_ alone—although not to the same extent as observed in the RPMI control medium (without lCFS_Pa_) (**Figures 8B, C**).

These findings confirm that *P. aeruginosa* secretes molecules with fungistatic activity against *C. auris* and *C. albicans*. Furthermore, this inhibitory effect is modulated by iron availability.

## 4 Discussion

*C. auris* and *P. aeruginosa* are pathogens that have been extensively studied and are well known for their resistance to antimicrobials and disinfectants, as well as for their ability to persist in hospital environments and infect immunocompromised patients, particularly those receiving broad-spectrum antibiotic therapy (Du et al., 2020). Consequently, they are major contributors to HAIs.

Given their common characteristics, it is reasonable to hypothesize that these microorganisms may interact within a shared environment, potentially co-infecting or co-colonizing the same patient (Khan et al., 2020). Indeed, metagenomic studies of the skin microbiome of residents of a specialized mechanical ventilation unit revealed simultaneous colonization by *C. auris* and *P. aeruginosa* (Proctor et al., 2021). Similarly, another study involving nursing home residents showed that the skin—particularly the nostrils and inguinal folds—served as a reservoir for *C. auris* and *P. aeruginosa* (Proctor et al., 2025). These findings raise important questions about how these pathogens interact with each other and the host microenvironment.

In our study, two widely used techniques for *in vitro* research were employed to investigate the interaction between *P. aeruginosa* and *C. auris*: the spot-on-the-lawn assay and liquid medium co-culture. The spot-on-the-lawn method, initially introduced by Harris et al. (1989), is commonly used to evaluate antagonistic activity between microorganisms (Sun et al., 2016; Acurcio et al., 2020; Bastos et al., 2022; Peres-Emidio et al., 2023). This technique measures the size of inhibition zones formed by substances produced by a bacterium. Before introducing a fungal species, the bacterium is inactivated using chloroform vapor, thus ensuring that the fungus interacts solely with the substances diffused in the medium, rather than with live bacterial cells. In contrast, the liquid co-culture method allows direct interaction between live microorganisms, enabling mutual sensing and physical contact.

Our results demonstrated that, regardless of the technique used, *P. aeruginosa* consistently suppressed *C. auris* growth, as it does with *C. albicans*. Moreover, this inhibitory effect was also observed across multiple *C. auris* strains, indicating a conserved interaction. The co-culture experiment also revealed that yeast growth inhibition began early (between 8 and 12 hours) and persisted for up to 72 hours. Upon contact with *P. aeruginosa*, *C. auris* cells appeared unable to proliferate but remained viable. This was confirmed by fluorescence microscopy, which showed that, although the number of *C. auris* cells during co-culture reduced to 36% compared to monoculture, about 97% remained viable even after 72 hours of co-cultivation with the bacterium. These results support the idea that *P. aeruginosa* probably induces a fungistatic effect against *C. auris*.

Several studies have described the interaction between *P. aeruginosa* and *Candida* spp. as generally antagonistic to the yeast species (Ader et al., 2008; Lindsay et al., 2014; Fourie and Pohl, 2019; Peres-Emídio, 2020), aligning with the findings of this study. This antagonism may result from nutrient competition or the secretion of molecules that impair yeast growth (Fourie and Pohl, 2019).

To investigate the hypothesis that growth inhibition results from nutrient competition, co-cultivation assays were performed in various nutrient-rich media. However, inhibition levels remained relatively unchanged, suggesting that adding glucose or other nutrients does not alter the antagonistic effect. Besides, the spot-on-the-lawn assay demonstrated that *C. auris* growth was inhibited even in the absence of live bacterial cells, supporting the hypothesis that *P. aeruginosa* secretes compounds capable of suppressing fungal growth. This was further reinforced by experiments using concentrated (lyophilized) *P. aeruginosa* cell-free supernatant (lCFS), significantly reducing fungal proliferation when reconstituted in RPMI. In contrast, non-lyophilized CFS had no significant impact, indicating that the concentration of these secreted molecules is critical to achieving the antifungal effect against *C. auris*.

*P. aeruginosa* is particularly known to inhibit *C. albicans* growth by sequestering iron through siderophores, whose production is upregulated in the presence of the yeast (Fourie et al., 2016; Kramer et al., 2020). Iron is an essential micronutrient for the growth and metabolism of many pathogens, serving as a cofactor in key metabolic processes (Bastos et al., 2020; Proctor et al., 2025), including oxidative phosphorylation and electron transport within the respiratory chain (Caza and Kronstad, 2013). Its significance is underscored by its universal necessity for the survival and proliferation of living organisms (Fourie and Pohl, 2019). *P. aeruginosa*, in particular, has a strong dependence of iron for its growth and efficiently competes with other organisms for it (Zhao and Yu, 2018). Moreover, *P. aeruginosa* PA14 produces metabolites that restrict iron uptake by other microorganisms, such as *A. fumigatus* (Proctor et al., 2025) and *C. albicans* (Rahme et al., 1997; Grainha et al., 2020).

In order to investigate whether a mechanism similar to that observed in *C. albicans* also applies to *C. auris*, co-cultivation and lCSF_(Pa)_ inhibition assays were performed in RPMI medium supplemented with different concentrations of FeSO_4_. Supplementation reduced the inhibitory effect of *P. aeruginosa* or lCSF_(Pa)_ on *C. auris*, although it was not sufficient to completely restore fungal growth. Notably, in the presence of lCFS_(Pa)_, growth restoration occurred in a dose-dependent manner relative to FeSO_4_.

These results are in line with findings reported by Kovács et al. (2025), who demonstrated that exposure of *C. auris* to N-(3-oxododecanoyl)-L-homoserine lactone (HSL)—a key quorum-sensing molecule produced by *P. aeruginosa*—inhibited fungal growth and altered gene expression. Specifically, HSL downregulated genes involved in iron homeostasis, potentially leading to reduced intracellular iron levels in *C. auris*. These data support the hypothesis that *P. aeruginosa* secretes factors capable of disrupting iron metabolism in *C. auris*, thereby contributing to growth inhibition (Ader et al., 2008). Additionally, another study reported that *P. aeruginosa* produces other molecules with anti-*C. auris* activity, such as fluopsin C (Spoladori et al., 2023), a broad-spectrum antibiotic that contains a copper ion chelated by two thiohydroxamate groups (Patteson et al., 2021). While the exact mechanism of fluopsin C against *C. auris* remains unclear, it may also interfere with ion homeostasis (Spoladori et al., 2023), further impairing yeast growth.

Although iron plays a crucial role in restoring the growth capacity of *C. auris*, other extracellular factors produced by *P. aeruginosa* may also contribute to the antifungal effect. For example, phenazines, such as pyocyanin, are toxic to fungi and other eukaryotes due to their redox-active properties, which can induce the formation of reactive oxygen species (ROS) (Mudaliar and Prasad, 2024). Additionally, rhamnolipids might be involved in the inhibitory effect, as they are known to suppress β-1,3-glucan synthase, an important enzyme involved in the biosynthesis of fungal cell walls (Briard et al., 2017). Further experiments are needed to elucidate the precise mechanisms through which *P. aeruginosa* inhibits *C. auris*.

In conclusion, our results demonstrates that *P. aeruginosa* exhibits a significant ability to inhibit *C. auris* growth, similar to its effect on *C. albicans*. This inhibitory activity does not seem to depend on the inoculum size and incubation time, pointing to a fungistatic mechanism consistent across different culture media. The inhibition is likely mediated by chemical substances secreted by *P. aeruginosa*, and iron availability emerges as a contributing factor in modulating this interaction.

Furthermore, our study highlight the potential of substances secreted by *P. aeruginosa* as antifungal agents against *C. auris*, contingent upon their concentration. Our results provide a foundation for future research to elucidate the chemical composition and mechanisms of action of these bioactive compounds. Furthermore, they contribute to a broader understanding of microbial interactions and their potential implications for hosts during co-colonization or infection.

## Data Availability

The datasets presented in this study can be found in online repositories. The names of the repository/repositories and accession number(s) can be found below: https://repositorio.ufrn.br/bitstream/123456789/60104/1/Interacaometabolicaentrefungo_Macedo_2024.pdf.
